# Relatives’ and Intensive Care Unit Personnel’s Perspectives of Care in Organ Donation: Protocol for a Multiple Methods Study

**DOI:** 10.2196/55643

**Published:** 2024-11-11

**Authors:** Kathe B Meyer, Gudrun Rohde, Gro Frivold

**Affiliations:** 1 Department of Transplant Medicine Oslo University Hospital Oslo Norway; 2 Department of Health and Nursing Sciences University of Agder Kristiansand Norway

**Keywords:** organ procurement, qualitative research, public health, patient-reported outcome, organ donation, organ care, perspectives, donors, intensive care personnel, Scandinavia, donor relatives, family satisfaction, descriptive analysis, comparative analysis, organ harvesting

## Abstract

**Background:**

In organ donation from deceased donors, the interaction between the donor’s relatives and intensive care personnel is an important factor. The organ donation (OD) process is complex, and patients’ relatives play a vital role. Intensive care professionals need knowledge about how relatives perceive and experience the process to create a caring environment and support them throughout. Therefore, this collaborative project aims to explore both relatives’ and intensive care personnel’s perspectives of care in deceased organ donation in Scandinavia.

**Objective:**

This study aims to (1) investigate donor relatives’ satisfaction and ICU personnel’s perception of their own professional competence and (2) explore donor relatives’ and ICU personnel’s experiences in the OD process to design for care and support in OD.

**Methods:**

This protocol outlines a Scandinavian (Norway, Sweden, and Denmark) project, including 4 work packages. Work package 1 started in 2023 with the translation and validation of the Family Satisfaction in the Intensive Care Unit questionnaire into a Danish version and the translation of the Professional Competence in Organ Donation Questionnaire into a Swedish and Danish version. A cross-sectional survey measuring Scandinavian relatives’ perception of support in and satisfaction with the organ donation process and a cross-sectional survey measuring Scandinavian intensive care personnel’s competence in organ donation are the foundation for work package 2 (2024). The data from both surveys will be analyzed using descriptive and comparative analysis. The results will inform the interview guides in qualitative studies (work packages 3 and 4). Participants in the quantitative study will be invited to participate in in-depth interviews. In work package 3, in-depth interviews will be conducted to illuminate relatives’ experiences in the organ donation process. The interviews will be analyzed using thematic analysis, according to Braun and Clarke. In work package 4 (2025-2026), 1 qualitative design study will be conducted to illuminate ICU personnel’s experiences. Furthermore, the results from work packages 2 and 3 will inform the development of specific programs for care, support, and communication in the organ donation process.

**Results:**

The project was funded by the Norwegian Organ Donor Foundation in 2022 and Scandiatransplant in 2023. The Norwegian Nurses Organisation supports the project by funding a PhD student. The PhD student was employed by the University in Agder in May 2024.

**Conclusions:**

This project will provide new knowledge that will assist us in designing and establishing programs for care, support, and donor relatives’ involvement in OD processes.

**International Registered Report Identifier (IRRID):**

PRR1-10.2196/55643

## Introduction

### Background

Organ donation (OD) is essential for organ transplantation, the lifesaving treatment for patients with terminal organ failure. However, worldwide, there is a gap between the need for organs and the number of deceased organ donors [1,2]. In 2023, there were 2210 new notifications on the transplant lists in Scandiatransplant, the organ exchange organization for the Nordic countries. Although 1982 people received an organ from a deceased donor, as many as 1627 people were still waiting for an organ by the end of the year. Many of the patients on transplant lists will either be withdrawn from the list due to deteriorating health or die while waiting for an organ [3]. The process of procuring organs from a deceased donor is complex, and patients’ relatives play a vital role [4-7]. It starts with the admittance of the patient to the emergency department, followed by failed treatment attempts and declaration of death in the intensive care unit (ICU), and finally, organ procurement in the operating theatre. In the months after the donation, donor relatives are offered a follow-up conversation initiated by the ICU personnel [6,8-10].

Whether to become a deceased organ donor is an individual decision [11-14]; yet, in most cases, ICU personnel ask the deceased’s relatives for an interpretation of the deceased’s preference and decision regarding organ donation. As OD often is the result of sudden and unexpected death, relatives are not prepared. They find themselves in a devastating situation facing the sudden death of a loved one, and they may have many thoughts regarding the usability of the body, the purpose of the donation, and the notions of meaning and hope in this tragedy [5,15,16]. Hence, the ICU personnel’s competence in communication and the OD process is crucial when there is a potential deceased donor (DD) in the ICU [17-20]. To gain more knowledge about relatives’ needs and the ICU personnel’s competence in OD processes, we conduct a Scandinavian collaborative project in Norway, Sweden, and Denmark. These neighboring countries are cooperating partners in Scandiatransplant, have a common history, and the health care system and culture are fairly similar. This project involves users who have personal experiences they want to share. One of them, Anne Stine, lost her husband in an accident. At the ICU, the question about organ donation was raised. She says: “Even if we knew Niels’ wishes related to organ donation, it was crucial that the health personnel had a humble, respectful approach to the topic. One of the most important issues is enough time to be able to get used to the idea that someone close to you has passed away. Time to take a decision. The way it is communicated can be decisive” [21].

Qualitative studies investigating relatives’ experiences with OD in general show that they may have difficulties understanding and dealing with the severity of the situation and that their relationship with the intensive care staff is of great importance [10,15,22-24]. Lack of comprehension can present a challenge, and understanding the process helps people reconcile with their decision [10]. Furthermore, the prolonged interventions necessary to enable OD can be misleading and make it difficult to understand brain death [17]. Relatives of ICU patients are at increased risk for long-term health impairments such as anxiety, depression, and complicated grief [25,26]. In the OD process, in which all treatment is deemed futile, and the patient becomes a potential DD, a lack of knowledge can make donation a fearful experience [27]. Hence, the ICU personnel have a multidimensional responsibility to create a caring environment and provide correct and comprehensive information to accommodate relatives’ understanding and to prevent psychological impairment. At the same time, this cooperating, caring relationship enables organ donation and ensures that there are organs available for patients waiting for organ transplantation [4,6,28].

### Organ Donation Methods

The OD process has traditionally taken place after the patient has been declared dead by neurological criteria, what is known as donation after brain death (DBD) [1]. In these cases, the patient is declared dead while the heart and lungs are still functioning, and vital organs can be preserved by medical treatments. Hence, the patient’s family members may perceive that their loved one is still alive [17]. To meet the excessive demand for donor organs, organ donation after circulatory death (DCD) has been introduced to complement DBD. The former can be performed in a controlled context (controlled donation after circulatory death [cDCD]) when treatment is deemed futile, and death cannot be determined by neurological criteria [1,2,29]. In these cases, following the decision to withdraw life-sustaining therapies, the family members witness the controlled death of the patient [22]. Thereafter, the family must leave the room quite rapidly, and the organ donation procedure is performed. This OD method is now available in Scandiatransplant’s member countries, Norway, Sweden, Denmark, and Finland [13,30]. There is a significant need for further knowledge about relatives’ experiences related to both methods of OD.

### Relatives’ Satisfaction With Care and Decision-Making

The quality of health care services must be evaluated using ongoing measurement of satisfaction [31,32], which is lacking in organ donation research. Previous studies [32-35] have investigated relatives’ satisfaction with care and decision-making support in the ICU, with results showing that there is potential for improvement. When it comes to the process of OD, relatives are the unique source of information about satisfaction in end-of-life care in the ICU. Studies investigating donor families’ psychological well-being [36-38] have shown that donation may have a beneficial effect on the bereavement process, whereas negative feelings related to organ donation might increase the symptoms of posttraumatic stress [38]. None of the studies described above focuses on how the process in the ICU is perceived by the relatives. In Belgium, Poppe et al [39] investigated the quality of communication and emotional support during the organ donation process. Most relatives reported receiving emotional support; however, the support decreased during the process.

### ICU Personnel’s Competence in the OD Process

ICU personnel, both physicians and nurses, are responsible for identifying a potential donor and partaking in donor management and care for the next of kin. However, OD is a rare event, and in line with international research [40-44], scarce experience and insufficient competence in approaching next of kin and medical aspects regarding OD have been reported in previous Norwegian studies [45-47]. Internationally, education programs have been developed to enhance the quality of the communication and emotional support offered during the donation process [48,49]. In Norway, Sweden, and Denmark, there are similar interdisciplinary programs where ICU personnel are educated in the OD process. The education programs include donor management and communication; however, as far as we know, their effect has not been evaluated. All 3 countries have donor-responsible doctors and nurses at their donor hospitals, and in some regions or hospitals, there are specialized nurses in organ donation. Any hospital may initiate the donor process; however, in Norway, organ retrieval is allowed only at hospitals the health authorities have certified.

### Needs Description

There is a lack of studies exploring experiences related to both DBD and cDCD, and few studies have investigated relatives’ perspectives on care and support during the organ donation process, including aftercare of relatives in general [50]. There is also a lack of knowledge about ICU personnel’s professional competence and experiences related to the 2 donation methods. To our knowledge, no previous research focusing on donor relatives’ or ICU personnel’s experiences and perceptions of care, decision-making, and support across ICUs in Scandinavia exists, despite the longstanding cooperation in organ exchange within Scandinavian countries. The cDCD method of organ donation has recently been reintroduced in the Nordic countries as a complement to DBD. Consequently, we have a unique opportunity to explore relatives’ experiences in both groups. This project will allow us to identify aspects that might influence relatives’ perceptions of the OD process. These aspects will form the basis of future work to enhance ICU personnel’s competence in care and support for relatives when OD is an option. Therefore, there is a need to assess perceptions of and satisfaction with care, decision-making, and support during the organ donation process in Scandinavian ICUs.

### Aim and Research Questions

#### Overview

The overall aim of the project is to explore relatives’ and ICU personnel’s perspectives of care and support in OD in Scandinavia by increasing the acknowledgment of family involvement and designing programs for care and support in OD, both DBD and cDCD, including donor relatives’ aftercare.

#### Research Questions

1. What is donor relatives’ satisfaction with care, decision-making, and support in the OD process?
2. What are donor relatives’ experiences in the OD process?
3. What is ICU personnel’s perception of their own professional competence in the OD process?
4. What are ICU personnel’s experiences in the OD process?

## Methods

### Overview

**Figure 1:**
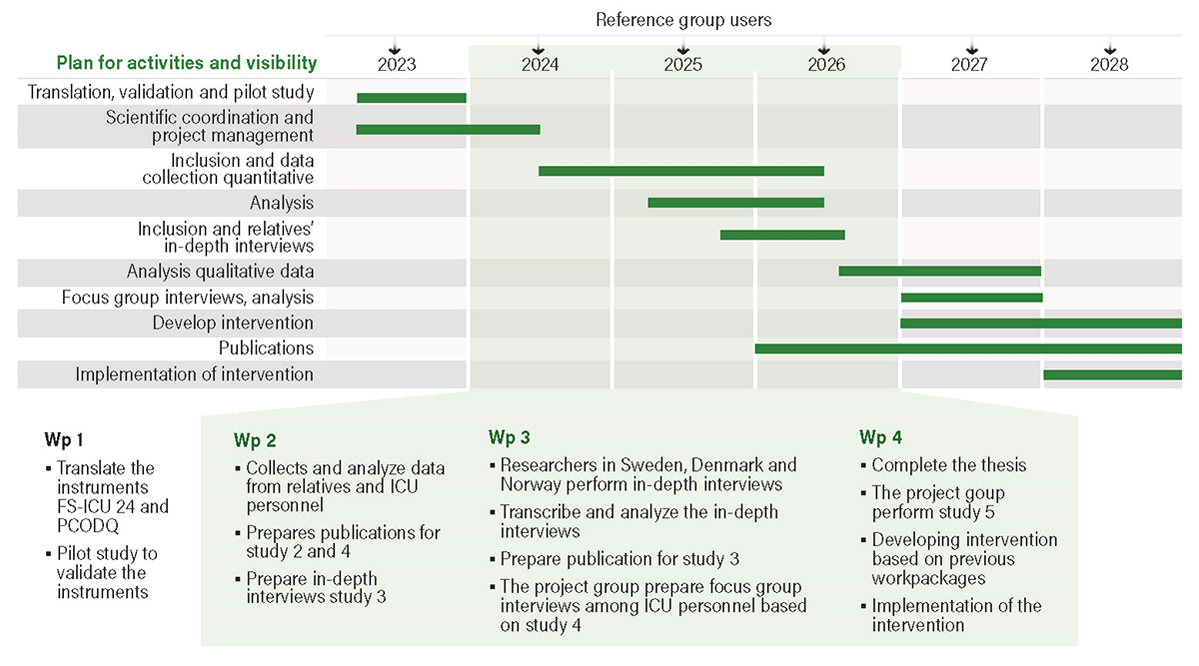
Work packages. Wp: work package; FS-ICU: Family Satisfaction in the Intensive Care Unit; PCODQ: professional competence in organ donation questionnaire; ICU: intensive care unit.

This project consists of 4 work packages and includes 2 quantitative studies to assess relatives’ and ICU personnel’s perceptions of the organ donation process and 2 qualitative studies to explore relatives’ and ICU personnel’s experiences and perspectives related to the 2 methods of OD. The studies will be the basis for designing and implementing follow-up programs to improve clinical practice in organ donation across Scandinavian countries (Figure 1). The project started in 2023 and will be completed in 2028.

### Work Package 1: Translation and Validation of Instruments (2023)

The translation process followed recognized guidelines for forward and backward translation [51,52]. A reference group reflects the perspectives of the donor relatives and of health care professionals in the ICU. The project group consists of members from the 3 countries. The Swedish and Danish research team members will act as gate openers in the clinical field. The Danish team performed the translation of the instrument Family Satisfaction in the Intensive Care Unit (FS-ICU) 24 into Danish and will test it in a pilot study (study 1). The Danish version of FS-ICU will be tested for content and internal consistency using Cronbach’s alpha during the spring of 2024 and will be published in a separate scientific paper. The project group members in Sweden and Denmark approved the translation of the instrument measuring ICU personnel’s competence in the respective languages. Validation will be performed accordingly.

### Work Package 2: Assessment of Relatives’ Perceptions of and Satisfaction With Care, Decision-Making, and Support, and ICU Personnel’s Professional Competence in the Organ Donation Process (2024-2025)

#### Overview

Assessment of donor relatives’ perceptions of care in and satisfaction with the OD process by using the FS-ICU 24 questionnaire developed by Heyland and Tanmer [32,33,53] will start in 2024 (study 2). Simultaneously, ICU personnel’s perception of their own professional competence in OD will be assessed (Study 4) by using the professional competence in organ donation questionnaire (PCODQ) developed by Meyer et al [47].

#### Study Design

Study 2: across-sectional survey to explore relatives’ satisfaction and perceptions of the OD process will be conducted consisting of the following information.

Demographics such as relatives’ nationality, gender, age, relation to the donor, and donation-specific questions.

The FS-ICU 24 questionnaire will assess donor relatives’ satisfaction [32]. The questions concern the perception of care, communication with the ICU staff, the quality of information, and perceptions of the decision-making process. The items are scored on a 5-point Likert scale from ranging from poor to excellent. The questionnaire was used in a Norwegian cross-sectional study [33] to measure satisfaction with care and decision-making in the ICU and psychometrically tested in a Norwegian context [54]. Even though the questionnaire was not developed to scope the donor relatives’ satisfaction we will use it in this project.

#### Setting and Recruitment

In Sweden and Denmark there are OD registries where the public may opt-in or opt-out, while in Norway a person may register their attitude on the national health website; however, it is not a formal OD registry. As the transplantation and organ donation acts in all 3 countries states that the deceased will should be followed, the relatives have an interpretative role in the OD. Former relatives involved in OD processes in a Scandinavian ICU in the last 2-24 months will be invited to participate. There are approximately 100-200 deceased donors a year in the respective countries. Relatives describe OD as a distinctive experience that does not fade over time. Hence, we anticipate that an inclusion period of 2-24 months will be sufficient. We will establish a network of ICU personnel to recruit eligible participants. Based on power calculation statistics, we will need a sample of 92 relatives from each country. Furthermore, to allow for dropouts, we will include an extra 20%. Thus, we aim to enroll 110 individuals from each country.

Relatives who were involved in a DBD or cDCD process for a close family member in an ICU in Norway, Sweden, or Denmark will be included. We will include relatives with experiences from DBD or cDCD in all 3 countries. They can be included even if the decision-making process resulted in no donation. Children younger than 18 years, relatives without the capacity to consent, and persons who are not able to understand Scandinavian languages will not be invited to participate.

#### Data Analysis

The FS-ICU scale will be recoded into scores ranging from 0 to 100, (0=poor, 25=fair, 50=good, 75=very good, and 100=excellent). Transforming item scores to a 0-100–point scale makes the values more meaningful and more appropriate for statistical analyses. We will perform descriptive and comparative analyses. Descriptive analyses will be generated for family members’ demographics and for scores on perceptions of and satisfaction with care. We will use multiple linear regression analysis to identify adjusted associations between care, communication support, and family satisfaction. The data will also be stratified by country to check for any differences among the 3 countries.

Study 4: across-sectional survey to explore ICU personnel’s perception of their competence in organ donation will be conducted consisting of the following information.

Background variables include profession, specialization, type of hospital, experience in OD, both care and communication and clinical experience in the ICU.

ICU personnel’s professional competence in OD will be measured by the PCODQ [47]. The questionnaire consists of 32 items exploring the different dimensions of theoretical, practical, ethical, and socially mediated knowledge of professional competence on a 5-point Likert scale.

We also assess what they perceive as most important for developing their own professional competence in OD.

#### Setting and Recruitment

The network of ICU personnel will assist in recruiting eligible personnel. The ICUs vary in terms of size and site, such as university, regional, or local hospitals. ICU personnel at university and regional hospitals may have more experience in organ donation than those at local hospitals. The practice and approach may also vary between countries and hospitals. ICU physicians and nurses who have been involved in at least 1 OD will be recruited in ICUs in the 3 countries. They will receive an invitation letter, which will include information about the study and a consent form by email. Those who consent to participate will receive an electronic questionnaire.

#### Data Analyses

An independent samples *t* test will be used to test differences between ICU physicians and nurses. Regression analysis will be used to test potential associations between background variables and perception of professional competence. One-way ANOVA will be used to test potential differences between ICU personnel in the 3 countries. Cronbach α will be performed to test the validity of the Swedish and Danish versions of PCODQ.

### Work Package 3: Qualitative In-Depth Interviews to Gain Insight Into the OD Process From Relatives’ Own Perspectives (2025)

#### Overview

Study 3: to capture the relatives’ experiences in the OD process, we will use a qualitative design with in-depth interviews. In-depth interviews can provide rich information about the OD process from the relatives’ own perspectives. The results from the cross-sectional survey in Study 2 will allow us to identify areas that need further in-depth exploration, and a semistructured interview-guide will be developed based on previous studies [5,16] and the cross-sectional study.

#### Study Design

##### Recruitment

We will recruit participants among eligible relatives who accept an invitation attached to the survey (study 2). Approximately 6 or 7 participants from each country will be purposively selected based on the areas identified in Study 2 as needing further exploration. We will include 20 participants altogether.

##### Data Collection

Interviews with former donor relatives will be conducted in Norway, Sweden, and Denmark. The interviews will be conducted by the PhD student in Norway and by the core researchers in Sweden and Denmark. By interviewing relatives about their experiences during and after the OD process, themes and items found in the quantitative study (study 2) can be further explored, and new phenomena might be revealed. The interviews will be recorded by a recorder recognized for research and transcribed by native-speaking transcribers in Norway, by the PhD student, and in Sweden and Denmark by a professional service.

The time and place of the interviews will be chosen in accordance with the participants’ preferences.

##### Data Analysis

The qualitative data will be analyzed using a thematic analysis (TA) comprising an interpretative approach, according to Braun and Clarke [55]. The 6 steps of TA are (1) get familiar with the data by reading, rereading, and searching for patterns across the dataset; (2) coding; (3) searching for themes across the codes; (4) reviewing the themes; (5) defining and naming the themes; and finally (6) relating the results to the research question and the existing literature. The TA was chosen as it is flexible and allows for the search of patterns and meaning across the dataset.

The NVivo software (Lumivero) will be used for qualitative analysis.

### Work Package 4: Exploring ICU Personnel’s Experiences and Designing a Plan to Implement the New Knowledge Obtained in the Previous Stages in Intensive Care Settings (2026-2027).

#### Overview

Studies 2-4 will be completed, and the project group will conduct a qualitative study to gain insight into ICU personnel’s experiences in the OD process (study 5).

Study 5: qualitative study to explore ICU personnel’s experiences in DBD and cDCD processes. We will use focus group interviews to gain a variety of perspectives on OD. In contrast to individual interviews, focus group interviews use group dynamics to acquire qualitatively good data about the participants’ experiences and opinions. Furthermore, the advantage of using focus groups is that the participants consider their own views in the context of others [56].

#### Study Design

##### Recruitment

Both ICU physicians and nurses from the respective countries who participated in the cross-sectional survey and agreed to participate in the qualitative study will be invited to participate.

##### Data Collection

The results from the 3 previous studies will inform the development of a semistructured interview guide. A Norwegian researcher will moderate the focus group interviews in Norway and participate in the focus group interview, while a Swedish and Danish researcher will moderate in the respective countries.

##### Data Analysis

The project group will perform a thematic analysis [55] to identify themes and patterns.

### Implementation

The knowledge gained from studies 2-5 will form the foundation of specific follow-up programs for care, support, and communication throughout the OD process, starting during the ICU stay and decision-making process and ending with an aftercare intervention.

### Ethical Considerations

#### Overview

The project has ethical approval from the Regional Ethical Committee (REK sør-øst C 502704) and Sikt (445633) [57]. To ensure appropriate ethical considerations throughout the study phases, we will follow the World Medical Association (WMA) Declaration of Helsinki [58]. This project involves relatives who might be vulnerable. The participants will receive oral and written information about the project. The researchers will act with sensitivity when it comes to disclosure of aspects of lived experience that would normally be kept private [59]. Throughout the project phases, we will be special attentive to the risks of psychological burdens and possible re-traumatization. If there is a need for further psychological support, the project members will establish an appropriate support team.

Clinical staff members will provide information sheets to relatives who are potential participants, and the survey will be sent directly by the clinic using their registered contact information. We will use web survey software to ensure the participants’ anonymity. Relatives and ICU personnel who wish to participate in the interview studies will return their replies and completed questionnaires. All participants will be able to withdraw their consent to participate at any time during the process, and we will ensure confidentiality and anonymity in reporting throughout the research process. No names of individuals or hospitals will be exposed in the interview transcriptions or reporting. The transcriptions will be treated confidentially and be kept under lock and key at the University in Agder. The project partners have clear guidelines and regulations for collecting, storing, and processing research data. All data will be collected according to the European General Data Protection Regulation [60]. The University in Agder and project partners will establish data-sharing agreements as needed. All data will be treated in strict adherence to the regulations of the data protection officers at the University in Agder and project partners.

#### Contingency Plan

In order to meet any problems, we have established an expert group consisting of professionals in organ donation or intensive care from the 3 countries in addition to the research group.

## Results

The project was funded by the Norwegian Organ Donor Foundation in February 2022 and Scandiatransplant in March 2023. The Norwegian Nurses Organisation decided to support the project by funding a PhD student for 4 years. The PhD student was employed by the University in Agder on May 1, 2024. The recruitment will start during the autumn of 2024.

## Discussion

### Principal Findings

This research is novel because no previous research has focused on relatives’ experiences and perceptions of care, decision-making, and support or ICU personnel’s perceptions of their professional competence in OD processes across ICUs in Scandinavia, despite the long-existing cooperation in organ exchange. By conducting this study, aspects that might influence relatives’ and ICU personnel’s perceptions of OD processes can be identified. These aspects will form the basis of future work to improve care and support for relatives when OD is an option.

The cDCD method has recently been reintroduced in the Nordic countries as a complement to DBD. Consequently, we have a unique opportunity to explore the experiences of relatives in both groups. In addition, the perspectives on processes that did not lead to organ donation need to be illuminated.

The new knowledge obtained through the 2 studies reflecting the relatives’ perspectives will inform efforts to increase professional competence in the organ donation process and to prepare ICU personnel for meeting relatives in the future. Based on this knowledge, and the knowledge we will obtain about ICU personnel’s perception of their own professional competence and their experiences in OD, we plan to organize regional workshops to develop systems that can ensure appropriate support and aftercare for donor relatives [16,18,33,40,47]. A more uniform approach towards OD seems to be beneficial [9,18,40]. Furthermore, it seems important that the relatives of the donors understand the OD process. Support during the process and aftercare, where the relatives have the opportunity to ask questions and receive information both about the OD process and the result of the donation, may reduce a potential burden [5,7,10,17,25]. Relatives are important ambassadors; their communication about the topic, their experiences, and their stories will influence their social surroundings. These experiences can be decisive for others’ responses to the question of organ donation [16]. To share this knowledge and raise public awareness, the Division of Communication at the University in Agder will offer coaching for publishing feature (op-ed) stories in the press [61].

### Strengths and Limitations

This project has several strengths; one important strength is that it will include donor relatives and ICU personnel from 3 Scandinavian countries. Another important strength is that the cross-sectional surveys will provide a broad understanding of relatives’ perceptions of and satisfaction with care and support, as well as of ICU personnel’s professional competence in the OD process. Using both quantitative and qualitative methods enables both broad and in-depth insight.

One limitation is the different ICU cultures and resources in the Scandinavian countries, which might be a challenge in recruiting participants on the one hand, but on the other hand, might be a strength in the interpretation of the results.

Both relatives and ICU personnel will be anonymous participants in the surveys, and the project members will not be active participants in the recruitment. This might be another limitation as there will be no control over who has been invited to participate or any possibility of a reminder.

### Conclusion

This project will provide new knowledge that will assist us in designing and establishing programs for the care of both potential donors and their relatives. Furthermore, support and donor relatives’ involvement in OD processes will be emphasized.

#### Patient and Public Involvement

To ensure the project’s relevance, 4 family members who have been involved in the organ donation process are included. Their contribution in planning during the different phases of the project is invaluable. They all have unique experiences that are essential in developing the interview guide for the in-depth interviews. Furthermore, their previous experiences as relatives of deceased organ donors will provide a different perspective than the researchers, and they will be involved in the analysis and interpretation of the results and the dissemination of the new knowledge.

An ICU physician and 1 ICU nurse from 2 donor hospitals in Norway will also participate in the reference group to provide the perspective of health professionals and contribute to the dissemination of new knowledge.

Another member of the reference group is a woman with a kidney transplant. She is a local team leader of the Norwegian Association for Kidney Disease and Transplantation. She has a broad network and provides yet another perspective to this research project.

#### Dissemination

The study results will be disseminated through publications in international peer-reviewed journals, presentations at national and international conferences, and in forums for intensive care health care professionals.

## Abbreviations

cDCD: controlled donation after circulatory death
DBD: donation after brain death
DCD: donation after circulatory death
DD: deceased donor
FS-ICU: Family Satisfaction in the Intensive Care Unit
ICU: intensive care unit
OD: organ donation
PCODQ: professional competence in organ donation questionnaire
TA: thematic analysis
WMA: World Medical Association

## Data Availability

The datasets used and/or analyzed during this study are not publicly available due to General Data Protection Regulation laws but are available from the corresponding author on reasonable request and with permission from the Norwegian Centre for Research Data.

## References

[1] Lomero M, Gardiner D, Coll E, Haase-Kromwijk B, Procaccio F, Immer F, Gabbasova L, Antoine C, Jushinskis J, Lynch N, Foss S, Bolotinha C, Ashkenazi T, Colenbie L, Zuckermann A, Adamec M, Czerwiński J, Karčiauskaitė S, Ström H, López-Fraga M, Dominguez-Gil B, European Committee on Organ Transplantation of the Council of Europe (CD-P-TO) (2020). Donation after circulatory death today: an updated overview of the European landscape. Transpl Int.

[2] Manara A, Procaccio F, Domínguez-Gil Beatriz (2019). Expanding the pool of deceased organ donors: the ICU and beyond. Intensive Care Med.

[3] Scandiatransplant (2024). Transplantation and waiting list figures. Scandiatransplant figures Aarhus, Denmark 2023.

[4] Gripewall E, Kerstis B, Bjorling G, Fagerstrom L, Mattsson J, Widarsson M, Nyholm L (2022). Intensive care nurses’ experiences of caring during the organ donor process in Sweden – a qualitative study. Int J Caring Sci.

[5] Jensen AMB (2016). "Make Sure Somebody Will Survive from This": Transformative Practices of Hope among Danish Organ Donor Families. Med Anthropol Q.

[6] Meyer K, Bjørk IT (2008). Change of focus: from intensive care towards organ donation. Transpl Int.

[7] Salvesen PH, Bergland A (2022). «Å avslutte et kapittel» – intensivsykepleieres erfaringer med etterlattesamtaler etter organdonasjoner. inspira.

[8] Avilés Lissette, Kean S, Tocher J (2022). Edgework emotion management: A constructivist grounded theory of organ donation nurses' experiences and practices. J Clin Nurs.

[9] Domínguez-Gil B, Coll E, Elizalde J, Herrero JE, Pont T, Quindós B, Marcelo B, Bodí M A, Martínez A, Nebra A, Guerrero F, Manciño J M, Galán J, Lebrón M, Miñambres E, Matesanz R, ACCORD-Spain study group. (2017). Expanding the Donor Pool Through Intensive Care to Facilitate Organ Donation: Results of a Spanish Multicenter Study. Transplantation.

[10] Berntzen H, Bjørk I T (2014). Experiences of donor families after consenting to organ donation: a qualitative study. Intensive Crit Care Nurs.

[11] Helse- og omsorgsdepartementet (2015). Lov om donasjon og transplantasjon av organ, celler og vev (transplantasjonslova).

[12] Socialstyrelsen (2022). New regulations concerning organ-preserving treatment and other provisions Stocholm: Socialstyrelsen.

[13] Socialstyrelsen (2023). Donation process. [Donationsprocessen] Stockholm, Sweden 2023.

[14] Sundhedsstyrelsen (2023). Organ donation. Legislation and organisation [Organdonation. Lovgivning og organisering] Århus, Denmark Sundhetsstyrelsen.

[15] Fridh I, Forsberg A, Bergbom I (2009). Close relatives' experiences of caring and of the physical environment when a loved one dies in an ICU. Intensive Crit Care Nurs.

[16] Kentish-Barnes N, Cohen-Solal Z, Souppart V, Cheisson G, Joseph L, Martin-Lefèvre L, Si Larbi AG, Viquesnel G, Marqué S, Donati S, Charpentier J, Pichon N, Zuber B, Lesieur O, Ouendo M, Renault A, Le Maguet P, Kandelman S, Thuong M, Floccard B, Mezher C, Duranteau J, Azoulay E (2019). Being convinced and taking responsibility: a qualitative study of family members' experience of organ donation decision and bereavement after brain death. Crit Care Med.

[17] Gyllström Krekula L, Forinder U, Tibell A (2018). What do people agree to when stating willingness to donate? On the medical interventions enabling organ donation after death. PLoS One.

[18] Hulme W, Allen J, Manara AR, Murphy PG, Gardiner D, Poppitt E (2016). Factors influencing the family consent rate for organ donation in the UK. Anaesthesia.

[19] Hvidt NC, Mayr B, Paal P, Frick E, Forsberg A, Büssing A (2016). For and against Organ Donation and Transplantation: Intricate Facilitators and Barriers in Organ Donation Perceived by German Nurses and Doctors. J Transplant.

[20] Jawoniyi O, Gormley K, McGleenan E, Noble HR (2018). Organ donation and transplantation: Awareness and roles of healthcare professionals-A systematic literature review. J Clin Nurs.

[21] Sarpebakken J (2022). Det var vesentlig at vi hadde snakket om det: Landsforeningen for nyresyke og transplanterte.

[22] Syversen TB, Sørensen DW, Foss S, Andersen MH (2018). Donation after circulatory death - an expanded opportunity for donation appreciated by families. J Crit Care.

[23] Jensen AM (2011). Orchestrating an exceptional death [Dissertation].

[24] Kesselring A, Kainz M, Kiss A (2007). Traumatic memories of relatives regarding brain death, request for organ donation and interactions with professionals in the ICU. Am J Transplant.

[25] Fumis RRL, Ranzani OT, Faria PP, Schettino G (2015). Anxiety, depression, and satisfaction in close relatives of patients in an open visiting policy intensive care unit in Brazil. J Crit Care.

[26] Kentish-Barnes N, Lemiale V, Chaize M, Pochard F, Azoulay E (2009). Assessing burden in families of critical care patients. Crit Care Med.

[27] AlHajri L, AlHebsi A, AlSuwaidi M (2021). How context affects people's willingness to register for the deceased organ donation programme. BMC Public Health.

[28] Mills L, Koulouglioti C (2016). How can nurses support relatives of a dying patient with the organ donation option?. Nurs Crit Care.

[29] Foss S, Nordheim E, Sørensen DW, Syversen TB, Midtvedt K, Åsberg A, Dahl T, Bakkan PA, Foss AE, Geiran OR, Fiane AE, Line PD (2018). First Scandinavian Protocol for Controlled Donation After Circulatory Death Using Normothermic Regional Perfusion. Transplant Direct.

[30] Giske L, Solberg B, Tranvåg EJ (2019). Organ donation with the use of normothermic regional perfusion in patients who die after cardiac and respiratory arrest after withdrawal of life-sustaining treatment.

[31] Stricker KH, Kimberger O, Schmidlin K, Zwahlen M, Mohr U, Rothen HU (2009). Family satisfaction in the intensive care unit: what makes the difference?. Intensive Care Med.

[32] Heyland DK, Tranmer JE, Kingston General Hospital ICU Research Working Group (2001). Measuring family satisfaction with care in the intensive care unit: the development of a questionnaire and preliminary results. J Crit Care.

[33] Frivold G, Slettebø Å, Heyland DK, Dale B (2018). Family members' satisfaction with care and decision-making in intensive care units and post-stay follow-up needs-a cross-sectional survey study. Nurs Open.

[34] Heyland DK, Dodek P, Rocker G, Groll D, Gafni A, Pichora D, Shortt S, Tranmer J, Lazar N, Kutsogiannis J, Lam M, Canadian Researchers End-of-Life Network(CARENET) (2006). What matters most in end-of-life care: perceptions of seriously ill patients and their family members. CMAJ.

[35] Heyland DK, Rocker GM, Dodek PM, Kutsogiannis DJ, Konopad E, Cook DJ, Peters S, Tranmer JE, O'Callaghan CJ (2002). Family satisfaction with care in the intensive care unit: results of a multiple center study. Crit Care Med.

[36] Tavakoli SAH, Shabanzadeh AP, Arjmand B, Aghayan SHR, Nozary Heshmati B, Emami Razavi S, Bahrami Nasab H (2008). Comparative study of depression and consent among brain death families in donor and nondonor groups from March 2001 to December 2002 in Tehran. Transplant Proc.

[37] Ahmadian S, Khaghanizadeh M, Zarghami MH, Khaleghi E, Ebadi A (2018). Tools for the Measurement of Psychological Aspects of Organ Donation among the Families of Brain-dead People. Int J Organ Transplant Med.

[38] Merchant SJ, Yoshida EM, Lee TK, Richardson P, Karlsbjerg KM, Cheung E (2008). Exploring the psychological effects of deceased organ donation on the families of the organ donors. Clin Transplant.

[39] Poppe C, Akum S, Crombez G, Rogiers X, Hoste E (2019). Evaluation of the quality of the communication and emotional support during the donation procedure: The use of the donor family questionnaire (DFQ). J Crit Care.

[40] Hancock J, Shemie SD, Lotherington K, Appleby A, Hall R (2017). Development of a Canadian deceased donation education program for health professionals: a needs assessment survey. Can J Anaesth.

[41] Babaie M, Hosseini M, Hamissi J, Hamissi Z (2015). Knowledge, Attitude and Practice of Nurses Regarding Organ Donation. Glob J Health Sci.

[42] Flodén A, Persson LO, Rizell M, Sanner M, Forsberg A (2011). Attitudes to organ donation among Swedish ICU nurses. J Clin Nurs.

[43] Fernandes Vasconcelos T, Gonçalves Menegueti M, Corsi C, Michelon-Barbosa J, Sato L, Basile-Filho A, Becari C, Dantas RAS, Auxiliadora-Martins M (2022). Assessment of physicians' knowledge about brain death and organ donation and associated factors. Medicine (Baltimore).

[44] Frenette AJ, Williamson D, Weiss MJ, Rochwerg B, Ball I, Brindamour D, Serri K, D'Aragon F, Meade MO, Charbonney E (2020). Worldwide management of donors after neurological death: a systematic review and narrative synthesis of guidelines. Can J Anaesth.

[45] Bratberg L (2015). Holdninger til organdonasjon blant leger og sykepleiere: Høgskolen i Oslo og Akershus.

[46] Eide H, Foss S, Sanner M, Mathisen JR (2012). Organ donation and Norwegian doctors' need for training. Tidsskr Nor Laegeforen.

[47] Meyer K, Bjørk IT, Eide H (2012). Intensive care nurses' perceptions of their professional competence in the organ donor process: a national survey. J Adv Nurs.

[48] Muthny FA, Wiedebusch S, Blok GA, van Dalen J (2006). Training for doctors and nurses to deal with bereaved relatives after a sudden death: evaluation of the European Donor Hospital Education Programme (EDHEP) in Germany. Transplant Proc.

[49] Blok GA, Morton J, Morley M, Kerckhoffs CCJM, Kootstra G, van der Vleuten CPM (2004). Requesting organ donation: the case of self-efficacy--effects of the European Donor Hospital Education Programme (EDHEP). Adv Health Sci Educ Theory Pract.

[50] Kerstis B, Widarsson M (2020). -Relatives' Experiences When a Family Member Is Confirmed Brain Dead and Becomes a Potential Organ Donor-A Literature Review. SAGE Open Nurs.

[51] Maneesriwongul W, Dixon JK (2004). Instrument translation process: a methods review. J Adv Nurs.

[52] Tsang S, Royse C, Terkawi A (2017). Guidelines for developing, translating, and validating a questionnaire in perioperative and pain medicine. Saudi J Anaesth.

[53] Wall RJ, Engelberg RA, Downey L, Heyland DK, Curtis RJ (2007). Refinement, scoring, and validation of the Family Satisfaction in the Intensive Care Unit (FS-ICU) survey. Crit Care Med.

[54] Dale B, Frivold G (2018). Psychometric testing of the Norwegian version of the questionnaire Family Satisfaction in the Intensive Care Unit (FS-ICU-24). J Multidiscip Healthc.

[55] Braun V, Clarke V (2022). Thematic Analysis : A Practical Guide.

[56] Patton MQ (2015). Qualitative Research & Evaluation Methods : Integrating Theory and Practice. 4th ed.

[57] UNINETT (2022). Sikt - The knowledge sector's service provider.

[58] World Medical Association (2013). World Medical Association Declaration of Helsinki: ethical principles for medical research involving human subjects. JAMA.

[59] Beauchamp TL, Childress JF (2019). Principles of Biomedical Ethics. Eighth edition.

[60] Rosemary J (2020). Data Protection Law and Practice. Data Protection Law and Practice.

[61] Symvoulakis EK, Markaki A, Anyfantakis D, Rachiotis G (2018). Organ Donation Awareness: Rethinking Media Campaigns. Int J Health Policy Manag.

